# *In vivo* efficacy of alkaline peroxide tablets and mouthwashes on *Candida albicans* in patients with denture stomatitis

**DOI:** 10.1590/S1678-77572010000300017

**Published:** 2010

**Authors:** Altay ULUDAMAR, Yasemin Kulak ÖZKAN, Tanju KADIR, Ismail CEYHAN

**Affiliations:** 1 DDS, MSc, PhD, Private Dental Clinic, Kavaklıdere, Ankara, Turkey.; 2 Professor, Head of Prosthetic Dentistry at the University of Marmara, Faculty of Dentistry, Istanbul, Turkey.; 3 Professor, Head of Oral Microbiology at the University of Marmara, Faculty of Dentistry, Istanbul, Turkey.; 4 MSc, PhD, Microbiologist, Vice President, Ministry of Health, Refik Saydam National, Public Health Agency, Ankara, Turkey.

**Keywords:** Complete dentures, Denture cleansers, Denture stomatitis, *Candida albicans*

## Abstract

**Objective:**

Effective cleaning of dentures is important for the maintenance of good oral hygiene for denture stomatitis patients. The *in vivo* efficacy of three different brands of alkaline peroxide tablets (Polident, Efferdent, and Fittydent) and two mouthwashes (CloSYS II and Corsodyl) to eliminate *Candida albicans* on dentures was evaluated in this in vivo study.

**Material and methods:**

Ninety denture wearers with clinical evidence of denture stomatitis were randomly divided into 5 test groups and 1 control group. Each group was further divided into three subgroups in which the dentures were subjected to 15-, 30-, and 60-min disinfection procedures. The dentures of each test group were treated with one of the cleaners, while those of the control group were treated with distilled water. Swab samples from the palatal surfaces (2 cm x 2 cm template delimited area) of the upper dentures were obtained before and after 15, 30, and 60 min periods of cleaner use and examined mycologically.

**Results:**

The reduction in the number of colony-forming units (CFU) of *C. albicans* before, and after 15, 30, and 60 min of use of CloSYS II and Corsodyl was significantly greater than that of the control group (p<0.05). Moreover, there was no statistically significant difference (p>0.05) among Polident, Efferdent and the control group in any of the treatment periods. Dentures treated with Fittydent appeared to have a significantly greater reduction in the number of *Candida spp.* only after 60 min of treatment.

**Conclusions:**

The results of this study showed that the use of mouthwashes significantly reduced the number of microorganisms on dentures.

## INTRODUCTION

The presence of microbial film on the surface of maxillary dentures is an important etiologic factor in denture stomatitis^1,3,4,6-8,13,20,22,32,32,38,40^. Denture base acrylic resin is easily colonized by oral endogenous bacteria and *Candida spp*., and eventually by extraoral species such as *Staphylococcus* spp. or members of enterobacteriaceae. This microbial reservoir can be responsible for denture-related stomatitis and aspiration pneumonia, a life-threatening infection, especially in geriatric patients^18^.

Oral and denture hygiene of dependent elderly individuals is extremely poor^4,33,34,39^. Elderly people living in shelters and especially for handicapped denture wearers, denture cleaning is a common problem^17,19,29,33,39^. Many modalities for delivering oral care have been suggested in the literature. Studies comparing the efficacy of the proposed denture-cleansing techniques, either mechanical or chemical, have used a variety of methods to evaluate biofilm control^2,5,11,12,16,18,20,21,22-28,30,31,35,36^.

Soap is one of the auxiliary agents that can be used^37^. Previous data have shown that brushing with dentifrice is one of the most common methods of denture hygiene and specific pastes can be employed too^15,34^. Clinical studies regarding the effectiveness of soap, as an isolated method, have not been extensively reported. The effectiveness of soap is still contradictory^34^. Effective biofilm removal requires a degree of manual dexterity that is often lacking, especially among elderly patients^16,30^. The use of chemical denture cleaners can produce more effective results, especially in geriatric patients and in people who have problems with wearing dentures^29^. Chemical denture cleaners are classified into various groups such as alkaline peroxides, alkaline hypochlorite, acids, disinfectants, and enzymes^10,17^.

Recently, mouthwashes have been used for cleansing dentures^5^. Use of mouthwash is a good habit for everyone in order to enjoy optimal dental health. For general use, anti-caries and mouth refresher mouthwash is recommended. Chlorhexidine gluconate is an antiseptic and disinfectant agent, which is active against various bacteria, viruses, bacterial spores, and fungi. These include the *C. albicans* which causes thrush infection in the mouth, and bacteria that may infect mouth ulcers or other mouth sores, e.g. after dental surgery^5,9,10,14^. The clinical and microbiological efficacy of chlorine dioxide (ClO_2_) as a topical antiseptic and disinfectant agent also used for the treatment of chronic atrophic candidiasis in geriatric patients has been assessed^25^.

The general impression is that the available chemical cleaners are mostly effective on denture microorganisms^6,10,28,33^. However, no study has assessed clinically the efficacy of short-term use of these chemical denture cleaning agents. Therefore, the *in vivo* efficacy of three different brands of denture cleaners and two mouthwashes to eliminate *C. albicans* on dentures was investigated in this study.

## MATERIALS AND METHODS

### Subjects

In this study, 90 complete denture wearers who had generalized simple denture stomatitis, had no systemic disease and were wearing their present dentures for around 3 years. In accordance with the health care policy in Turkey, patients under social security scheme can only have their dentures replaced after 3 years. Therefore, the majority of patients selected for this study have been wearing their dentures for 3 years on average. Demographic details and full medical and dental history were obtained from each participant. Based on the information from health history and initial clinical examination, 90 patients (47 females and 43 males; age range: 45 to 75 years; mean age: 60.8 ± 15 years) with positive diagnosis of generalized denture stomatitis were included in this study. The clinical condition of palatal mucosa was recorded using the Budtz-Jorgensen and Bertram^6^ (1970) scale. All patients’ dentures were first cleaned in an ultrasonic cleaner for 5 min and then polished for 3 min using abrasives. The patients were advised not to clean their dentures for 3 days to standardize this study. When the patients came back for recall, a quantitative microbiological measurement was performed to establish a baseline value for the presence of *C. albicans*.

Swabs were taken from the palatal surface of the upper denture according to a 2 cm x 2 cm template delimiting the area to be swabbed. Swabs were cultured in Sabouraud medium containing 0.005% chloramphenicol and 0.04% cycloheximide. Candidal colony count was carried out after 48 h incubation at 37°C in aerobic conditions. *C. albicans* was differentiated from the other species by its production of filaments and its ability to grow on corn meal agar.

The patients were divided into 5 test groups and 1 control group of 15 subjects each. each group was further divided into three subgroups in which the dentures were subjected to 15-, 30-, and 60-min disinfection procedures. In the test groups, the dentures were disinfected with CloSYSII (Portola Plaza Dental Group, Mission Viejo, CA, USA), Corsodyl (GlaxoSmithKline Consumer, Health Group, Oakville, Ontario, Canada), Polident (GlaxoSmithKline Consumer Health Group, Oakville, Ontario, Canada), efferdent (Pfizer Consumer Health Care, Scarborough, Ontario, Canada) and Fittydent (Mag Hoeveler Co., Geinberg, Germany), respectively. The sixth group’s dentures were used as a control and kept in distilled water. Denture cleaners used in this study are shown in Figure 1.

**Figure 1:** Denture cleaners used this study

**Cleanser**	**Manufacturer**	**Content**
CloSYS II Oral Spray (C)	Portola Plaza Dental Group	Chlorine dioxide
Corsodyl Oral Spray (CO)	GlaxoSmithKline Consumer Health Group	0.2% chlorhexidine gluconate
Polident (PO)	GlaxoSmithKline Consumer Health Group	Carbon dioxide producers that contain citric acid, sodium bicarbonate and potassium monosulphate (pH 7.0 )
Efferdent (EFF)	Pfizer Consumer Health Care	Carbon dioxide producers that contain citric acid, sodium bicarbonate and potassium monosulphate (pH 7.5)
Fittydent (FITT)	Mag.Hoeveler & Co. Gmbh	Whitening power of baking soda and peroxide

The dentures were then treated in one of the following ways: *Mouthwashes*: CloSYSII was sprayed on the palatal surface of the upper denture (10 times and 5 cm away from the denture; 1 spray= 150 micl). Corsodyl was sprayed on the palatal surface of the upper denture (12 actuations 5 cm away from the denture; 1 actuation approximately 0.14 mL). For both products, 5 dentures were allowed to sit on the bench for 15 min, 5 dentures for 30 min, and 5 dentures for 60 min after being sprayed. *Effervescent tablets*: The dentures were placed in 200 cc of sterile distilled water with the respective denture cleaner. Five dentures were allowed to soak for 15 min, 5 dentures for 30 min, and 5 dentures for 60 min.

In the control group, sets of 5 dentures were soaked in distilled water for the same times as described above.

After the disinfection procedures, the dentures were immersed in sterile distilled water for 3 min and then swab samples were taken in the same way by the same investigator after treatment. The samples were mixed by using a vortex mixer at maximal setting for 30 s and ten-fold serial dilutions up to 10^–5^ were obtained in saline. Portions (0.1 mL) of dilutions were spread onto Sabouraud Dextrose (Delta Medical and Chemical materials Trading, Istanbul, Turkey) agar medium and plates were incubated for 48 h at 37°C. Plates with 100-300 colonies were then selected for colony enumeration and the number of colony-forming units (CFU) per cm^2^ were calculated^4,5^.

*C. albicans* isolates were identified using germ-tube test, chlamydospore formation on corn-meal agar and API 20C AUX (BioMerieux Vitek, Étoile, France) system.

### Statistical Analysis

**Figure 2:**
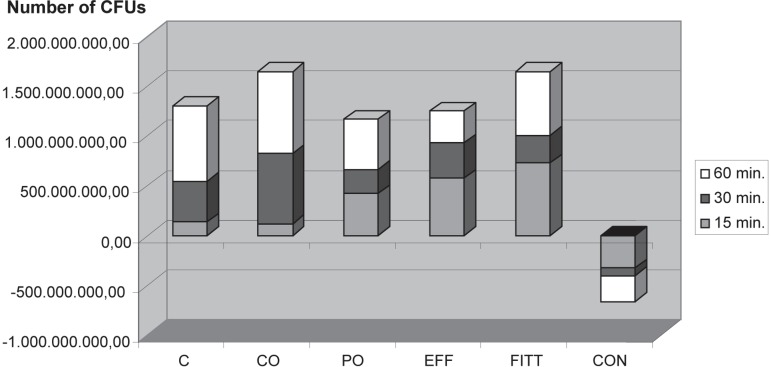
Number of Colony-Forming Units (CFU) at baseline minus number of CFUs 15, 30 and 60 minutes

The differences in the number of CFU of microorganisms before and after the three treatment times (15, 30, and 60 min) were examined to assess the effect of the cleansers relative to baseline (Figure 2). As this variable was not normally distributed, the natural logarithm, exponential square root, and rank of these differences were also determined. Four criteria for normality were examined: the median, the coefficient of skewedness, the coefficient of kurtosis, and the p value of the Kolmogorov goodness of fit for normality. The rank of the difference was better in producing nearly normal distributions, so it was used in the statistical parametric test (analysis of variance or ANOVA). The rank represents the position of each observation after sorting the variables by value. A general linear model was used to appraise differences in efficacy between cleansers. For these analyses, the mean of the difference in the number of CFU before and after each evaluation period was estimated. The distributions of these differences were not normal, so ranks were determined. In the statistical analysis, the mean of the differences in ranks for each period (baseline-15 min, baseline-30 min, and baseline-60 min) was used to test differences in efficacy between cleansers. In this multivariate analysis encompassing all study periods, the significance was analyzed with adjustment by difference of treatment, the treatment sequence, and the variance between study periods, and the variance between subjects.

## RESULTS

The results of the study are presented in Table 1. The cleaners are listed in order from most effective to least effective in their ability to kill and/or remove C. *albicans* from dentures worn by the patients in this study. The column labeled "mean percent reduction in CFU" indicates the mean decrease (or increase) in the number of CFU from the baseline swabs to swabs taken 15, 30, and 60 min after the disinfection procedures. The distilled water control did not produce a statistically significant decrease in the number of *C. albicans* from the baseline to the treated times.

**Table 1:** Mean difference * and Standard Deviation (SD) in number of Colony-Forming Units (CFU) of *Candida spp. *over the evaluation periods of 15, 30 and 60 min.

**Treatment group**	**15 min**	**30 min**	**60 min**
	**Mean difference (SD)**	**Mean difference (SD)**	**Mean difference (SD)**
			
CloSYSII	135.973.640,000	409.599.740,000	762.000.000,000
	(104.553.996,332)	(723.844.524,149)	(457.951.962,546)
			
Corsodyl	117.993.300,000	707.999.824,000	822.000.000,000
	(35.637.501,557)	(600.890.801,660)	(708.886.450,710)
			
Polident	423.761.200,000	244.747.060,000	503.957.306,000
	(435.114.370,500)	(425.339.479,977)	(490.961.285,036)
			
Efferdent	576.414.000,000	355.827.600,000	321.996.242,000
	(714.145.845,665)	(583.784.621,503)	(446.566.872,609)
			
Fittydent	733.054.400,000	276.215.280,000	641.974.418,000
	(624.820.079,636)	(461.957.246,754)	(524.735.767,607)
			
Control (water)	-322.000.000,000	-80.000.000,000	-262.200.000,000
	(464.133.601,455)	(186.815.416,923)	(507.768.845,047)

* Number of CFU at baseline minus number of CFU after the 15-, 30- and 60-min treatment times. Positive values represent a reduction and negative values an increase in the number of CFU relative to baseline.

All materials tested in this study showed a reduction in the number of *C. albicans* CFU. There was substantial variance among the 5 cleanser groups in the number of CFU of *C. albicans* at the end of various study periods (Table 2). In a multivariate analysis encompassing all study periods, dentures treated with CloSYSII and Corsodyl appeared to have a significantly greater reduction in the number of *Candida spp*. than those treated with Polident, efferdent, or Fittydent. The rank of the differences in numbers of CFU of *C.albicans* before and after the use of CloSYSII (p=0.04) and Corsodyl (p=0.02) for the 15 min disinfection periods were significantly different.

**Table 2:** P values for the number of colony-forming units of *Candida spp* over the evaluation periods of 15, 30 and 60 min.

**Treatment group**	**15 min**	**30 min**	**60 min**
			
CloSYSII	0.04 *	0.03 *	0.02 *
Corsodyl	0.002 *	0.05 *	0.04 *
Polident	0.09	0.26	0.08
Efferdent	0.14	0.24	0.18
Fittydent	0.06	0.25	0.05 *
Control (water)	0.196	0.393	0.313

* Significant difference between baseline and the three treatment times.

The rank of the differences in numbers of CFU of *C. albicans* before and after the use of Polident, efferdent, and Fittydent for the 15 min disinfection periods were not significantly different (p>0.05). Dentures treated with Polident and efferdent had no significant reduction in the number of *C. albicans* for all study periods. Denture treated with Fittydent appeared to have a significantly greater reduction in the number of *Candida spp*. only after 60 min of treatment. In addition, the differences in the rank of the number of CFU of *Candida spp*. was associated with the variances between the study periods (F: 2.34, p=0.001) and with the variances between subjects (F: 2.25; p=0.01) and the treatment sequence (F: 1.64: p=0.04).

## DISCUSSION

It is well accepted that chemical disinfectants have some advantages over mechanical cleaning, such as effective disinfection and ease of use^23,27,31^. Bacterial and yeast biofilm on dentures is thought to be an important factor in the pathogenesis of denture stomatitis^2,7,8^. Schou, Wight and Cumming^39^ (1987) showed that 60% of elderly patients living in shelters had complete dentures that were not considered clean. They found that these elderly patients did not have a habit of cleaning dentures, and their reason for not cleaning was that they would have had to expend effort. Also, mechanical cleaning of dentures is found to be insufficient for reducing the number of microorganisms on dentures, and palate^30,37^. It is well accepted that chemical disinfectants have some advantages over mechanical cleaning, such as effective disinfection and ease of use^27,28,31^. However, some studies showed that not all disinfectants are effective on the most important microorganism acting on dentures, *C. albicans* ^9^. Therefore, it has been suggested that all disinfectants should be investigated under the same conditions *in vitro* and *in vivo*, in order to eliminate the conflict between the studies.

The efficacy of the materials and methods of denture cleaning has been evaluated by means of *in vitro* and *in vivo* models. In the clinical trials, one of the methods employed for biofilm quantification is the microbiological evaluation. Thus, it is important that the discussion of the results obtained take it into account. That is, the results must be discussed by considering the kind of the analysis employed. It will be relevant for comparison of the several studies reported in the literature. There is also much discussion on the methodology of the studies which are investigating the efficacy of the chemical cleansers. The *in vitro* part of the studies may show different results from the *in vivo* study because of the variation in soaking temperature, time, and variation of the operators^13^. After even 3 min, the effect of the spray was seen in the *in vitro* study, but the same effect was not found until 30 min in the *in vivo* study.

Alkaline peroxides are the most used denture cleansers^16,17,21,23,24,26^. In addition to their chemical effects, they remove stains mechanically to release oxygen. The final products of peracetic acid decomposition are water, oxygen, and carbon dioxide, which are biocompatible products present in nature. Although the manufacturer of the alkaline peroxide disinfectant used in the study recommends 15 min of immersion, immersion times of 30 and 60 min were also investigated because there are reports suggesting that this shorter time does not result in complete disinfection. The immersion times were tested in order to find out the time required for maximum (60 min) disinfection. The findings of this study showed that a 15 min immersion in alkaline peroxides was not sufficient to yield decontamination of the tested acrylic resins *in vivo*. It was discovered that all of the alkaline peroxide tablets reduced *C. albicans* colonies, but did not completely eliminate them.

Fittydent was found to be more effective than Polident and efferdent in reducing *C. albicans* after 60 min of immersion. Gornitsky, et al.^18^ (2002) found that dentures treated with Denture Brite (Crombie Kennedy Nasmark Inc., Ontario, Canada) appeared to have a significantly greater reduction in the number of *Candida spp*. than those treated with efferdent. No differences were observed between Denture Brite and Polident or between Polident and efferdent. They employed the mechanical method (brushing) concomitantly with the chemical method. Ghalichebaf, Graser and Zander^17^ (1982) stated that efferdent was a little more effective than Polident in reducing plaque, but less effective than other cleansers (Mersene; Colgate-Palmolive Co., Piscataway, NY, USA) and Clorox-Calgon (The Clorox Co., Oakland, Beecham Products, Pittsburgh, PA, USA) The methodology of quantification employed by Ghalichebaf, Graser and Zander^17^ (1982) was the biofilm staining. The dentures were worn by the patients before the tests for 24 h. McCabe, Murray and Kelly^23^ (1995) compared chemical and mechanical methods. These authors employed others chemical cleansers and evaluated the efficacy of the products in relation of reduction of the biofilm, stains and calculus. The validity of the results of these studies relates to methodology, the composition of cleanser, and the different disinfection times.

Corsodyl Spray containing 0.2% chlorhexidine gluconate was used as a mouth spray. This study results showed that Corsodyl completely eliminated *C. albicans*. There appear to be no previous study using Corsodyl as a denture cleanser in the literature. The benefit of this product may derive from the 0.2% chlorhexidine gluconate

Chlorhexidine gluconate, used as a mouthrinse or applied topically, has been shown to have a beneficial effect on bacterial colonization on the teeth and the development of gingivitis in humans^12,40^.There are several studies that state that chlorhexidine is effective on biofilm removal^5,9,10^.

CloSYSII oral spray was also used as a mouth spray and completely eliminated *C. albicans* in this study. There have been few studies using CloSYSII oral spray as a denture cleanser in the literature. The efficacy of topical 0.8% chlorine dioxide in the management of chronic atrophic candidiasis was demonstrated by Mohammad, et al.^25^ (2004), who stated that ClO_2_ provided a safe and clinically effective option in the management of chronic atrophic candidiasis.

The significant reduction in the number of *C. albicans* in this study suggests that the use of mouthwashes is a suitable method for cleaning dentures the general population. Further studies are needed to determine if daily use of mouthwashes can reduce the high prevalence of patients with denture stomatitis. This limited study investigated only the effect of cleansers on *C. albicans* reduction. Further research should be carried out to assess the bacteriostatic and bactericidal effects of the sprays on microorganisms.

## CONCLUSION

Within the limitations of this study, it may be concluded that mouthwashes present as an easy-to-use and effective treatment for *C. albicans* and can be used as a denture disinfectants.

## References

[1] Akpan A, Morgan R (2002). Oral candidiasis. Postgrad Med J.

[2] Andrucioli MC, Macedo LD, Panzeri H, Lara EH, Paranhos HF (2004). Comparison of two cleansing pastes for the removal of biofilm from
dentures and palatal lesions in patients with atrophic chronic
candidiasis. Braz Dent J.

[3] Arendorf TM, Walker DM (1987). Denture stomatitis: a review. J Oral Rehabil.

[4] Arikan A, Kulak Y, Kadir T (1994). Comparison of three different treatment methods for generalized
denture stomatitis. J Prosthet Dent.

[5] Arikan A, Kulak Y, Kadir T (1995). Comparison of different treatment methods for localized and
generalized denture stomatitis. J Oral Rehabil.

[6] Budtz-Jörgensen E, Bertram U (1970). Denture stomatitis. The aetiology in relation to trauma and
infection. Acta Odontol Scand.

[7] Budtz-Jörgensen E, Löe H (1972). Chlorhexidine as a denture disinfectant in the treatment of denture
stomatitis. Scan J Dent Res.

[8] Budtz-Jörgensen E (1974). The significance of Candida albicans in denture
stomatitis. Scand J Dent Res.

[9] Budtz-Jörgensen E, Stenderup A, Grabowski M (1975). An epidemiologic study of yeasts in elderly denture
wearers. Community Dent Oral epidemiol.

[10] Budtz-Jörgensen E (1979). Materials and methods for cleaning dentures. J Prosthet Dent.

[11] Chan EC, Iugovaz I, Siboo R, Bilyk M, Barolet R, Amsel R (1991). Comparison of two popular methods for removal and killing of bacteria
from dentures. J Can Dent Assoc.

[12] Chassot AL, Poisl MI, Samuel SM (2006). In vivo and in vitro evaluation of the efficacy of a peracetic
acid-based disinfectant for decontamination of acrylic resins. Braz Dent J.

[13] Consani RL, Mesquita MF, Arruda Nobilo MA, Henriques GE (2007). Influence of simulated microwave disinfection on complete denture base
adaptation using different flask closure methods. J Prosthet Dent.

[14] Davies RM, Jensen SB, Schiott CR, Löe H (1970). The effect of topical application of chlorhexidine on the bacterial
colonization of the teeth and gingiva. J Periodontal Res.

[15] Dikbas I, Koksal T, Calikkocaoglu S (2006). Investigation of the cleanliness of dentures in a university
hospital. Int J Prosthodont.

[16] Dills SS, Olsham AM, Goldner G, Brogdon C (1988). Comparison of the antimicrobial capability of an abrasive paste and
chemical-soak denture cleaners. J Prosthet Dent.

[17] Ghalichebaf M, Graser GN, Zander HA (1982). The efficacy of denture-cleansing agents. J Prosthet Dent.

[18] Gornitsky M, Paradisl I, Landaverde G, Malo AM, Velly AM (2002). A clinical and microbiological evaluation of denture cleansers for
geriatric patients in long-term care institutions. J Can Dent Assoc.

[19] Imsand M, Janssens JP, Auckenthaler R, Mojon P, Budtz-Jorgensen E (2002). Bronchopneumonia and oral health in hospitalized older patients. A
pilot study. Gerodontology.

[20] Keng SB, Lim M (1996). Denture plaque distribution and the effectiveness of a
perborate-containing denture cleanser. Quintessence Int.

[21] Kulak Y, Arikan A, Albak S, Okar I, Kazazoglu E (1997). Scanning electron microscopic examination of different cleaners:
surface contaminant removal from dentures. J Oral Rehabil.

[22] Lambert JP, Korlstad R (1986). Effect of benzoic acid detergent germicid on denture borne Candida
albicans. J Prosthet Dent.

[23] McCabe JF, Murray ID, Kelly PJ (1995). The efficacy of denture cleansers. Eur J Prosthodont Restor Dent.

[24] Minagi S, Tsunoda T, Yoshida K, Tsuru H (1987). Objective testing of the efficiency of denture-cleansing
agents. J Prosthet Dent.

[25] Mohammad AR, Giannini PJ, Preshaw PM, Alliger H (2004). Clinical and microbiological efficacy of chlorine dioxide in the
management of chronic atrophic candidiasis: an open study. Int Dent J.

[26] Moore TC, Smith De, Kenny GE (1984). Sanitization of dentures by several dentine hygiene
methods. J Prosthet Dent.

[27] Nakamoto K, Tamamoto M, Hamada T (1991). Evaluation of denture cleansers with and without enzymes against
Candida albicans. J Prosthet Dent.

[28] Nikawa H, Iwanaga H, Hamada T, Yuhta S (1994). Effects of denture cleansers on direct soft denture lining
materials. J Prosthet Dent.

[29] Padilha DM, Hugo FN, Hilgert JB, Dal Moro RG (2007). Hand function and oral hygiene in older institutionalized
Brazilians. J Am Geriatr Soc.

[30] Palenik CJ, Miller CH (1984). In vitro testing of three denture cleaning systems. J Prosthet Dent.

[31] Paranhos HF, Panzeri H, Lara EH, Candido RC, Ito IY (2000). Capacity of denture plaque/biofilm removal and antimicrobial action of
a new denture paste. Braz Dent J.

[32] Pereira-Cenci T, Del Bel Cury AA, Crielaard W, Ten Cate JM (2008). Development of Candida-associated denture stomatitis: new
insights. J Appl Oral Sci.

[33] Pietrokovski J, Azuelos J, Tau S, Mostavoy R (1995). Oral findings in elderly nursing home residents in selected countries:
oral hygiene conditions and plaque accumulation on denture
surfaces. J Prosthet Dent.

[34] Pietrokovski J, Tamari J, Mostavoy R, Levy F, Azuelos Y, Tau S (1990). Oral findings in elderly nursing home residents in selected
countries. Gerodontology.

[35] Pinto TM, Neves AC, Leão MV, Jorge AO (2008). Vinegar as an antimicrobial agent for control of Candida spp. in
complete denture wearers. J Appl Oral Sci.

[36] Rudd RW, Senia ES, McCleskey FK, Adams ED (1984). Sterilization of complete dentures with sodium
hypochlorite. J Prosthet Dent.

[37] Salles AE, Macedo LD, Fernandes RA, Silva-Lovato CH, Paranhos HF (2007). Comparative analysis of biofilm levels in complete upper and lower
dentures after brushing associated with specific denture paste and neutral
soap. Gerodontology.

[38] Samaranayake LP, MacFarlane TW (1980). An in-vitro study of the adherence of Candida albicans to acrylic
surfaces. Arch Oral Biol.

[39] Schou L, Wight C, Cumming C (1987). Oral hygiene habits, denture plaque, presence of yeasts and stomatitis
in institutionalized elderly in Lothian, Scotland. Community Dent Oral epidemiol.

[40] Sreenivasan PK, Mattai J, Nabi N, Xu T, Gaffar A (2004). A simple approach to examine early oral microbial biofilm formation
and the effects of treatments. Oral Microbiol Immunol.

